# Role of Contrast-Enhanced Ultrasound (CEUS) in Native Kidney Pathology: Limits and Fields of Action

**DOI:** 10.3390/diagnostics11061058

**Published:** 2021-06-08

**Authors:** Antonio Granata, Irene Campo, Paolo Lentini, Francesco Pesce, Loreto Gesualdo, Antonio Basile, Vito Cantisani, Matthias Zeiler, Michele Bertolotto

**Affiliations:** 1 Nephrology and Dialysis Unit, “Cannizzaro” Hospital, 95026 Catania, Italy; antonio.granata4@tin.it; 2 Department of Radiology, “Civile di Conegliano” Hospital, ULSS 2 Marca Trevigiana, 31015 Conegliano, Italy; 3 Nephrology and Dialysis Unit, San Bassiano Hospital, 36061 Bassano del Grappa, Italy; paolo.lentini@yahoo.it; 4 Nephrology, Dialysis and Transplantation Unit, Department of Emergency and Organ Transplantation, University of Bari, 70124 Bari, Italy; f.pesce81@gmail.com (F.P.); l.gesualdo@nephro.uniba.it (L.G.); 5 Radiology Unit I, Department of Medical Surgical Sciences and Advanced Technologies “GF Ingrassia”-University Hospital “Policlinico-San Marco”, University of Catania, 95123 Catania, Italy; basile.antonello73@gmail.com; 6 Department of Radiology, Policlinico Umberto I Hospital, “Sapienza” University of Rome, 00161 Rome, Italy; vito.cantisani@uniroma1.it; 7 Nephrology and Dialysis Unit, “Carlo Urbani” Hospital, 60035 Jesi, Italy; mrhz1@yahoo.com; 8 Department of Radiology, “Cattinara” Hospital, University of Trieste, 34149 Trieste, Italy; bertolot@units.it

**Keywords:** contrast-enhanced ultrasound, chronic kidney disease, ultrasonography, CEUS, renal disease, nephropathies, microbubbles

## Abstract

Gray scale ultrasound has an important diagnostic role in native kidney disease. Low cost, absence of ionizing radiation and nephrotoxicity, short performance time, and repeatability even at the bedside, are the major advantages of this technique. The introduction of contrast enhancement ultrasound (CEUS) in daily clinical practice has significantly reduced the use of contrast enhancement computed tomography (CECT) and contrast enhancement magnetic resonance (CEMR), especially in patients with renal disease. Although there are many situations in which CECT and CEMRI are primarily indicated, their use may be limited by the administration of the contrast medium, which may involve a risk of renal function impairment, especially in the elderly, and in patients with acute kidney injury (AKI) and moderate to severe chronic kidney disease (CKD). In these cases, CEUS can be a valid diagnostic choice. To date, numerous publications have highlighted the role of CEUS in the study of parenchymal micro-vascularization and renal pathology by full integration with second level imaging methods (CECT and CEMRI) both in patients with normal renal function and with diseased kidneys. The aim of this review is to offer an updated overview of the limitations and potential applications of CEUS in native kidney disease.

## 1. Introduction

The first guidelines on contrast-enhanced ultrasound (CEUS) date back to 2004 [1] and focused on hepatic disease, whereas non-hepatic applications were taken into account in 2008 [2]. The dramatic increase in non-hepatic applications for CEUS made it necessary to release dedicated guidelines in 2011 [3,4]. Ultrasound modes have been used as the first imaging method both for the native and transplanted kidneys, in patients with normal and with impaired renal function [5,6,7]. However, it is known that the use of B-mode and Doppler ultrasound alone represents a limitation in the study of kidney diseases, while the addition of contrast medium allows the display of vascular abnormalities unappreciated with conventional modes. CEUS of the kidney is off-label in Europe, but widely used for its effectiveness and safety, and recommended by European guidelines [3,4]. The purpose of this review is to offer an updated overview of potential applications and limitations of CEUS in native kidney disease.

## 2. Repeatability of CEUS

The question of the ability to perform CEUS in clinical practice is one of the most important obstacles in the application of this technique, even if the investigation is not technically difficult.

CEUS should be understood as an examination that is performed to complement the US examination in order to obtain additional data not obtainable with US methods or other imaging methods. For this reason, the execution of a CEUS requires adequate operator training. Using an expert operator, CEUS is an effective and repeatable imaging technique. Authors reported a very good inter-reader agreement in the characterization of renal cysts [8] and in the characterization of the renal infarct [9].

## 3. Tolerance and Safety

Echo-contrast media are normally well tolerated. To evaluate the safety of CEUS, we should consider not only the possible side effects related to the drug administration but also those caused by the interaction between microbubbles and ultrasounds. These are not excreted through the kidneys and can be safely administered to patients with renal insufficiency with no risk of contrast-related nephropathy or nephrogenic systemic fibrosis. Ultrasound contrast agents (UCAs) are the safest of the medical contrast agents. All the second-generation contrast agents, sulfur hexafluoride and perflubutane, are microbubbles composed of a low-solubility gas enveloped by a phospholipid shell.

As reported in the EFSUMB guidelines [3], UCAs have a low rate of anaphylactoid reactions compared to iodinated and gadoline contrast agents (0.014% vs. 0.035–0.095% vs. 0.001–0.01%). On the other hand, mild adverse reactions are more frequent (headache, nausea, chest pain and chest discomfort) and resolve spontaneously in a short time without consequence.

Usually, transitory pain at the injection point, altered taste or hypotension for vasovagal response are observed in less than 5% of subjects while rare major adverse reactions (ventricular arrhythmia) are related to the use of a high mechanical index and the consequent massive rupture of the bubbles in the tele-systolic phase. For this reason, the use of CEUS in cardiology requires particular attention [10]. On the other hand, the overall mortality rate for sulphur hexafluoride is 0.0006% in 14/2447083 exposed patients, confirming its safety against iodinated contrast agents (about 0.001%) and in these cases unfavourable underlying medical circumstances were found [3]. It is therefore advisable to pay attention to frail patients with unstable cardiovascular diseases in the days preceding CEUS.

The current guidelines advise to use a mechanical index lower than 0.2 kPa to minimize the rupture of the bubbles. Finally, current understanding cannot exclude subclinical damage and bio-effects at a distance from the use of the UCAs. Therefore, the use of these techniques should always be based on a correct evaluation of the clinical risk/benefit.

## 4. Limitations

In the study of renal pathology, the limits of CEUS are substantially superimposable to those found in ultrasound. In patients with anatomical kidney malformations, CEUS cannot always provide the desired information. Similar to MRI and CT for the detection of kidney tumors, an important limitation of CEUS is the difficulty of identification of both small lesions (<1 cm), especially if developing intra-parenchymally, and isoechoic with the surrounding parenchymal. CEUS is unable to provide a simultaneous view of both kidneys, as is the case in CT and MRI imaging.

## 5. Examination Technique and Normal Renal Parenchyma

**Figure 1:**
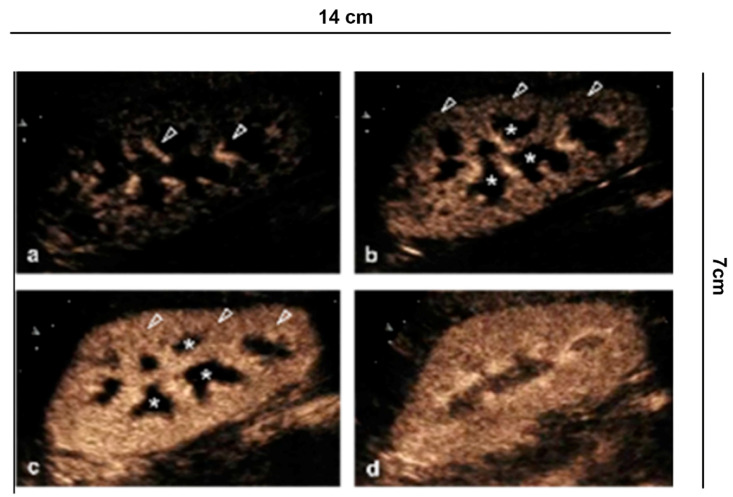
Renal vascularity with CEUS. (**a**) A few seconds after injection of the contrast medium, the microbubbles arrive in the arterial branches of the pedicle and in the branches of the interlobar artery (arrowhead). (**b**) After 15 s the cortical phase begins (arrowhead) without involving the medulla (asterisk). (**c**) After the completion of the cortical phase (arrowhead) comes the onset of the parenchymal phase (25 s–4 min) with progressive appearance of the enhancement of the external medulla (asterisk) and (**d**) subsequent involvement of the pyramids.

To perform a CEUS examination, it is necessary to have an ultrasound system equipped with microbubble-specific technology, capable of separating the signal coming from microbubbles (non-linear) from that of the stationary tissues (linear) [11]. To minimize artifacts and tissue signal, the lowest possible mechanical index is used, which depends on the type of equipment available. The contrast dosage ranges from 1.4 to 2.4 mL or less, depending on the ultrasound scanner, the patient’s habitus, and the depth of the lesion to be examined. All the CEUS images reported in this paper were obtained using an intravenous injection of 1.4–2.4 mL of microbubbles containing sulfur hexafluoride. The kidney is a remarkably vascularized organ, receiving approximately 21% of cardiac output. Segmental arteries branch from the main renal artery, and give rise to the lobar arteries, which in turn branch into the arciform and interlobar arteries. Then the contrast reaches the medullary circulation until reaching the pyramids (Figure 1) [3,4].

The vascular structures are rapidly represented, appreciating a fast enhancement of the renal parenchyma from the cortical surface to the portion of the cortico-medullary junction, best visible in the slow motion video. This is different in chronic renal insufficiency, where the enhancement is less intense and fades earlier.

## 6. Clinical Indications in the Native Kidney

There are numerous scenarios for which CEUS is indicated [1,3,4]. Microbubble injection often complements conventional ultrasound examination, but can also be considered as a second look examination in equivocal cases investigated with other imaging techniques (CECT and CEMRI).

### 6.1. Characterization of Cystic Lesions

The Bosniak classification has been the most widely adopted method to characterize cystic kidney lesions with good reliability regarding their nature [12]. The classification was first introduced for CECT, which is still considered the gold standard, with probability percentages of malignancy and benignity close to surgical ones [13,14]. The introduction of modern CT technologies did not significantly affect the performance of CECT for this task [15]. The Bosniak classification has been successfully applied also with CEUS, with a similar or better performance, compared to CECT [8,16]. Regardless of the technique used, the Bosniak classification system is intrinsically subjective, a problem well acknowledged since its introduction, which led to subsequent amendments of the scoring system [17]. A considerable effort to standardize the Bosniak scoring system and to improve interobserver agreement has been recently addressed by Silverman et al. for CECT and CEMRI, and by Cantisani et al. for CEUS [18,19]. Numerous aspects should be taken into account when complex renal cysts are scored in the US [18]. A multi-parametric approach is required. Scoring is not based on the size or shape of the cyst but only on the intra-cystic content, i.e., the presence or absence of septa, the number and the thickness of the septa, the wall thickness (more or less than 3 mm), and the presence of irregularities and of nodules.

**Figure 2:**
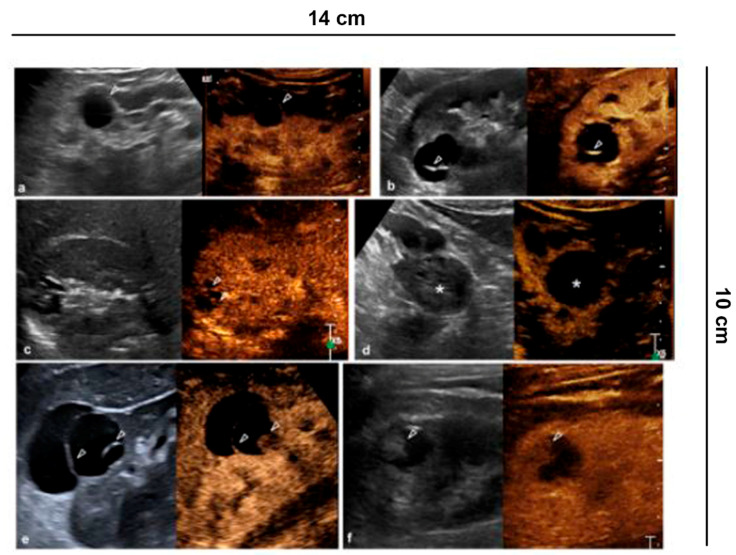
Characterization of cystic lesions. (**a**) Bosniak category I cyst (Simple benign cysts): B-mode US and CEUS show anechoic lesion without septa, calcification and wall irregularity; (**b**) Bosniak category II cyst (Minimally complex benign cysts): minimally complicated cyst with single calcific septum (arrowhead) on B-mode without recognizing enhancement on CEUS; (**c**) Bosniak category IIF cyst (Presumably benign, imaging surveillance is advised): on US B-mode, cyst with multiple thin septa, minimally thickened (2–3 mm), which on CEUS present minimal enhancement (arrowheads), without irregularities of both the wall and the septa; (**d**) Bosniak category IIF cyst (Presumably benign, imaging surveillance is advised): echogenic lesion with heterogeneous content inside (asterisk) on B-mode US, which does not show wall enhancement or septa on CEUS; (**e**) Bosniak category III cyst (Indeterminate lesions): on B-mode, cyst with two septa about 3 mm thick which have thickened, and irregular septa (arrowhead) on CEUS; (**f**) Bosniak category IV cyst (Likely malignant cystic tumors): cystic lesion with a wall nodule greater than 4 mm (arrowhead).

As complexity increases, the probability of being a malignant cyst grows. In the new EFSUMB classification, cystic tumors are included in category IV, or rather, probable malignant cystic tumors, according to histology. Cystic tumors comprise a diverse group of kidney lesions and can have variable biological profiles. Clearly, the US only identifies the presence of liquid and solid components, and CEUS identifies possible vascularization, referring the definition to histology (Figure 2).

### 6.2. Characterization of Indeterminate Lesions

**Figure 3:**
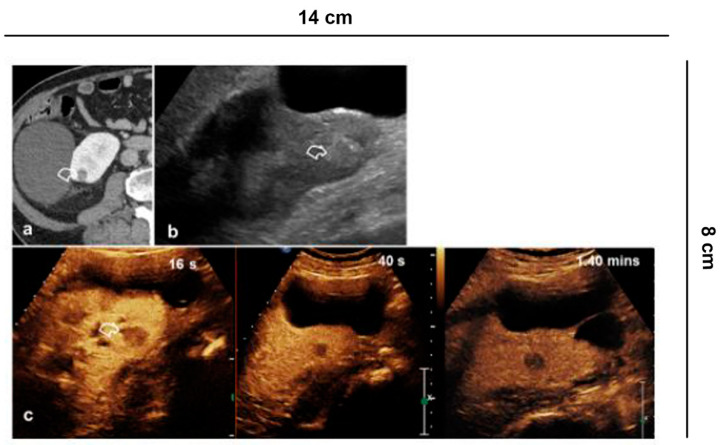
Indeterminate lesion. (**a**) 56-year-old man in follow-up for esophageal neoplasia. CECT acquired only in the portal phase. At the lower pole of the right kidney, there is a sharp-edged formation with equivocal enhancement (curved arrow); (**b**) B-mode US shows a hyperechoic solid lesion (curved arrow) which appears (**c**) hypovascularized in all vascular phases compared to the parenchyma on CEUS (curved arrow).

CEUS is recommended by the EFSUMB guidelines and by the American College of Radiology (ACR) to characterize incidental renal masses [3,4] Lesions with equivocal enhancement at CECT will be characterized as solid tumors or renal cysts with CEUS. Cysts are graded according to the Bosniak criteria [20] (Figure 3).

### 6.3. Characterization of Solid Renal Lesions

With the exception of angio-myolipomas with a visible amount of fat, differentiation of the different solid renal masses is problematic even at CECT and CEMRI. CEUS is limited for this task [21] and its use is not recommended by the EFSUMB guidelines for this purpose [3,4]. Several features, however, can help guess tumor histology at multiparametric US. Clear Cell Renal Cell Carcinoma (ccRCC) is usually hyper-vascular, with heterogeneous enhancement due to necrotic areas. However, differentiation from oncocytoma and lipid-poor angio-myolipomas remains problematic. Papillary Renal Cell Carcinoma (pRCC) is often hypo-vascular [22,23].

### 6.4. Differential Diagnosis between Solid Renal Masses and Pseudotumors

**Figure 4:**
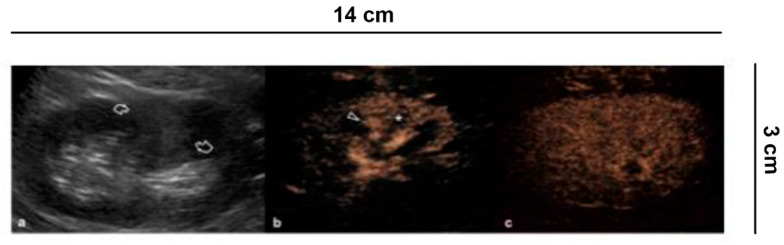
Differential diagnosis between solid renal masses and pseudo-tumors. (**a**) B-mode US shows a hyperechoic delimited area in the middle third of the left kidney as a possible solid lesion (curved arrows). (**b**) CEUS demonstrates, on the other hand, a normal cortical vascularization (arrowhead) with the intact medulla (asterisk) and with **(c)** the parenchymal phase preserved.

Anatomical variations can simulate a renal mass [24]. Among pseudo-tumors in particular, hypertrophy of the Bertin columns, persistence of fetal lobatures, and presence of humps can be confused with focal lesions requiring a CECT or CEMR study for characterization. CEUS is a valid alternative for a correct differential diagnosis [25,26,27]. In fact, pseudo-tumors will present the same enhancement characteristics as the renal parenchyma, without alterations of the vascular structures (Figure 4). In lesions iso-enhancing in all vascular phases, a situation that occurs in up to 5% of cases, differentiation from pseudo-masses is also possible combining grey-scale US and CEUS, since true lesions often present with different echogenicity, and pseudo-lesions usually display a medullary component which is later enhanced compared to cortex [26].

### 6.5. Follow-Up of Tumor Ablation

**Figure 5:**
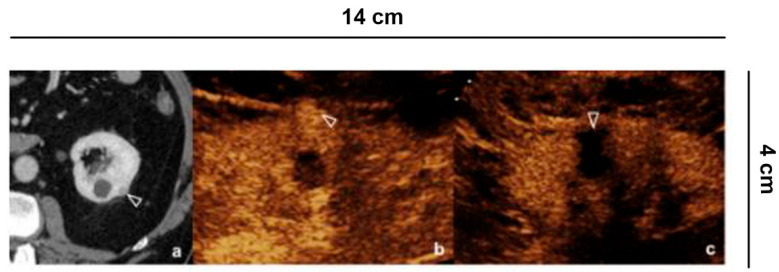
Follow up of microwave tumor ablation. (**a**) CECT shows a small renal lesion, partially exophytic, adjacent to a simple cyst, in the middle third of the left kidney; (**b**) Before the microwave treatment CEUS evidences a hyper-vascular, partially exophytic lesion (arrowheads); (**c**) After 12 h from the microwave ablation, CEUS shows an avascular area without recognizing the lesion (arrowheads), a sign of treatment success.

**Figure 6:**
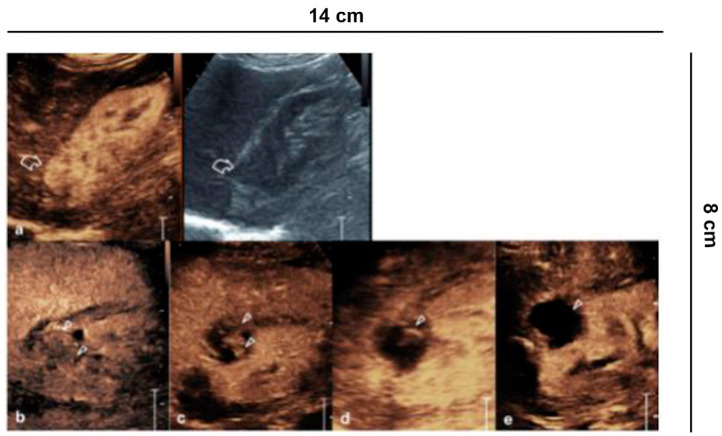
Ablative technique follow-up. (**a**) Before the cryo-ablative treatment, CEUS shows an iso-vascular, partially exophytic lesion at the upper pole of the right kidney (curved arrow). After treatment, CEUS shows diffuse and persistent intralesional vascularization (arrowheads) to 24 h (**b**); at 7 (**c**) and 14 days (**d**) there is a progressive reduction of vascularity; after one month (**e**) the lesion appears completely avascular, a sign of a successful treatment.

In accordance with the EAU guidelines [28], evaluation of ablative treatments of small kidney lesions requires a close monitoring of the ablated area, both to check for any complications and to assess the success of the procedure. CEUS has progressively taken a role in the follow-up, especially early after the procedure [29,30,31]. The treatment is considered successful if the lesion becomes completely avascular. This usually occurs early after radiofrequency and microwave ablation (Figure 5) [29,32], while it may take up to one month after cryoablation (Figure 6), requiring a tailored follow-up [30,31]. Therefore, an adequate knowledge of the type of ablative treatment allows a correct interpretation of the images, thus reducing the risk of misdiagnosis.

### 6.6. Complicated Pyelonephritis

**Figure 7:**
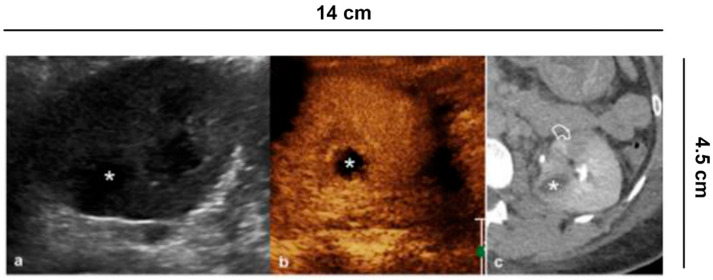
Complicated pyelonephritis. 53 year-old-woman with left flank pain and fever. (**a**) B-mode US appreciates inhomogeneous, hypoechoic area (asterisk); (**b**) CEUS performed in the same session confirms the presence of an avascular lesion with a peripheral rim, consistent with abscess (asterisk); (**c**) Urographic phase CECT, performed for the clinical worsening of the patient, confirms the presence of abscess (asterisk) and hypo-perfused area (curved arrow) at the lower pole of the kidney.

Pyelonephritis is a clinical diagnosis. Ultrasound is required to rule out obstructive causes. Other imaging such as CEUS or CECT are indicated in complicated pyelonephritis, or if clinical improvement is lacking at 72 h after medical treatment with antibiotics [33] (Figure 7). Ultrasound is not specific, and can be completely negative. Conversely, CEUS can visualize hypo-perfused areas of parenchyma up to true avascular, rounded areas, with possible peripheral rim, consistent with abscesses. Inflammatory areas are more visible in the parenchymal late phase [34]. In complicated pyelonephritis, CEUS can be used as a monitoring tool for therapeutic response.

### 6.7. Vascular Lesions

Renal vascular disease can involve vascular structures at all levels; in the clinical suspicion of renal infarction B-mode and Color Doppler US (CDUS) is always required, although they have limitations. In fact, this only provides in real time the average speeds coded with the color on the area of interest. The vascular representation of the more distal arterial branches is not always adequate, especially in elderly and in nephropathic patients.

**Figure 8:**
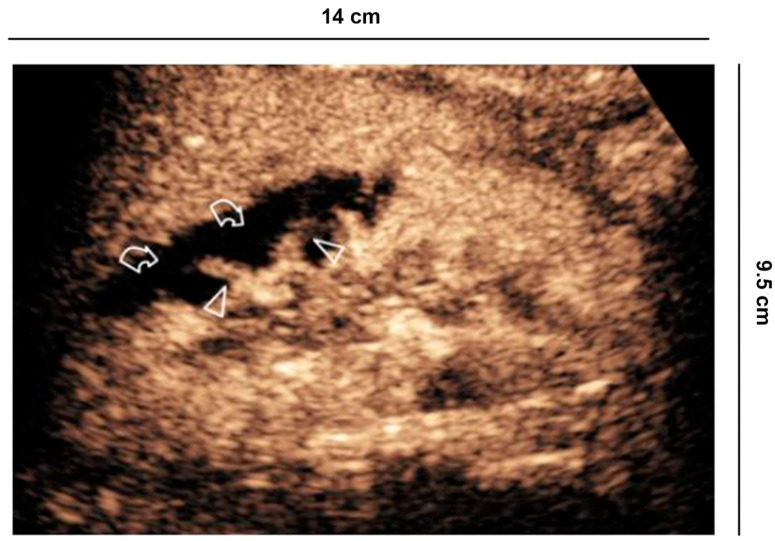
Vascular lesions. 56 years old man with atrial fibrillation shows creatinine elevation and right lower back pain. CEUS demonstrates focal acute cortical ischemia (curved arrow) and patent interlobar vessels (arrowheads).

Renal infarction (often in triangular form) or only cortical ischemia (absence of cortical interlobular vessels) (Figure 8) [9,35] are recognizable on the other hand with CEUS, which is able to enhance the lack of vascularization, and therefore repair the damage. The sensitivity of CEUS is quite similar to that of angiography and CT [9].

CEUS is increasingly being used for evaluation of organ perfusion and lesion characterization, as advocated by the EFSUMB guidelines. Specifically, the guidelines recommend CEUS for differentiating cortical necrosis from renal infarction. Real-time dynamic CEUS depicts microcirculation throughout the kidney and should therefore identify the reverse rim sign to diagnose acute cortical necrosis. The excellent spatial resolution of CEUS allows clear differentiation between renal infarction and cortical necrosis, which appears as non-enhancing cortical areas, and preserved hilar vascularity [35] between hypo-perfused and non-perfused areas is clear following ultrasound contrast agent administration.

**Figure 9:**
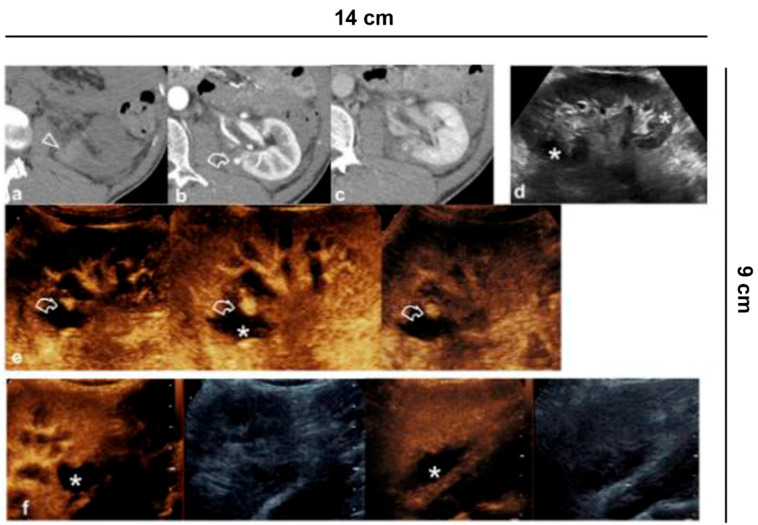
Vascular lesions. 35-year-old male patient with abdominal trauma treated conservatively. (**a**) CT without contrast medium shows parenchymal hyper-density from recent hematoma (arrowhead) at the lower pole of the left kidney; (**b**) A pseudoaneurysm (curved arrow) is recognized in the arterial phase, while in the (**c**) nephrographic phase the parenchymal contusion is more clearly recognized and the pseudoaneurysm is less visible; (**d**) B-mode US shows inhomogeneous echogenic areas (asterisks) corresponding to the contusions; (**e**) CEUS adequately shows all the vascular phases of the pseudoaneurysm (curved arrow) and the perirenal hematoma (asterisk); (**f**) Follow-up performed on 7th day.

CT remains the gold standard for the study of kidney trauma due to its panoramic view and its greater sensitivity compared to ultrasound. Thanks to its exclusively vascular nature, CEUS increases the sensitivity of US for the detection of traumatic lesions [36] allowing the study of all the vascular phases (arterial, venous and parenchymal) in real time, but above all it is useful in the follow-up in cases of conservative therapy (Figure 9) [37].

In addition, active bleeding can be recognized with some extravasated contrast medium bubbles and pseudoaneurysms can be detected. It should be remembered that the excretory system cannot be studied with CEUS and therefore when damage to the excretory tract is suspected, CT is mandatory.

#### 6.7.1. Renal Artery Stenosis

CDUS is considered the first-line imaging modality for the diagnosis of renal artery stenosis. This technique presents evident advantages in patients with chronic renal failure because it does not require nephrotoxic contrast agents. UCAs increase by 30-fold the intensity of the arterial Doppler signal, improve the visualization of the renal arteries and reduce the test duration and the number of non-diagnostic tests. Authors, in a study including 120 patients with 38 stenosed renal arteries, reported surprisingly good results [38]; sensitivity, specificity, PPV, NPV and accuracy were reported as being 100%, 84%, 0%, 80% and 94%, respectively, for CDUS compared with angiography. Recently, Cui et al. [39] have shown that CEUS is accurate in grading renal artery stenosis, and may represent the method of choice in diagnosing renal artery stenosis. Nevertheless, the use of CEUS in patients with renal artery stenosis is still debated and, in the near future, the application of new parametric software will allow for a quantitative analysis of contrast enhancement curves and a more accurate definition of renal perfusion.

#### 6.7.2. Renal Vein Thrombosis

In the differential diagnosis between renal cortical necrosis (RCN) and renal vein thrombosis (RVT), the Doppler US plays an important role. Spiesecke P. et al. [40] have shown that the resistive index (RI) is a very important distinguishing feature to separate RCN (RI not measurable due to hypoperfusion) and RVT (RI > 1). However, in unclear cases, the use of a contrast agent, which will demonstrate renal medulla enhancement only in patients with NCR and the absence of enhancement in patients with RVT, may be useful. CEUS increases diagnostic accuracy in the direct diagnosis of RVT, especially in cases of thrombosis secondary to neoplasia, providing useful information on any revascularization.

## 7. Potential Future Scenarios

CEUS now has an established role in renal imaging. New applications require development of innovative microbubbles. The commercially available microbubble contrast agents are characterized by a wide size distribution, typically ranging from 1–10 µm in diameter. Signal is produced by resonance, which depends on beam frequency and bubble diameter [41]. Contrast specific modes operate at a relatively narrow frequency bandwidth, which means only a small fraction of the microbubbles resonates and produce a signal. Bubble diameter also shows an inverse relationship with resonance frequency, which means that higher frequency imaging and improved spatial resolution is obtained if bubble diameter is reduced [41]. One option to improve contrast-specific imaging is the manufacturing of microbubble contrast agents with smaller diameter and narrower size distribution [42]. A dramatic increase in sensitivity has been documented in vitro for these bubbles [43].Moreover, these new agents may improve our ability to target microbubbles to specific endothelial cell ligands, transporting bioactive materials such as drugs or genes, and driving their behavior on specific targets triggering specific mechanisms, such as sonoporation [44]. This opens new perspectives in kidney diseases, especially for patients with AKI and CKD [45]. In a rat model of diabetic nephropathy, for instance, the therapeutic impact of combined cytotoxic T lymphocyte-associated antigen 4 immunoglobulin (CTLA-4-Ig) treatment and microbubble-mediated exposure has been investigated, showing that the effectiveness of this treatment improves following US microbubble exposure [46]. These applications are in an early pre-clinical phase, and future studies are required to investigate their full clinical potential.

## 8. Conclusions

CEUS is increasingly used to solve interpretative doubts arising with other methods. In fact, it can be considered both as a problem-solving technique in recognizing and distinguishing cystic vs. solid lesions, and as a technique to be used during follow-up in conservative treatments. The non-exposure to radiation and high safety profile allow it to be used even in rapid succession in cases of traumatic injuries treated conservatively, or in renal lesions requiring follow-up for prolonged periods, thus reducing the need for CECT and CEMRI scanning.

## Data Availability

The data presented in this study are available on request from the corresponding author.

## Abbreviations

AKI	acute kidney injury
ccRCC	clear Cell Renal Cell Carcinoma
CDUS	Color Doppler US
CECT	contrast-enhanced computed tomography
CEMRI	contrast-enhanced MRI
CEUS	contrast enhancement ultrasound
CKD	chronic kidney disease
CRF	chronic renal failure
CT	computed tomography
MRI	magnetic resonance imaging
pRCC	papillary Renal Cell Carcinoma
UCAs	ultrasound contrast agents
US	ultrasound
